# Ghost Melanoma: Endobronchial Melanoma of Unknown Primary Mimicking Primary Lung Cancer

**DOI:** 10.7759/cureus.116163

**Published:** 2026-09-13

**Authors:** Raihana Laamim, Samah Sadiki, Adil Zegmout, Hicham Souhi, Ismail Rhorfi

**Affiliations:** 1 Department of Pulmonology, Mohammed V Military Training Hospital, Rabat, MAR; 2 Department of Ophthalmology, Mohammed V Military Training Hospital, Rabat, MAR

**Keywords:** differential diagnosis, endobronchial melanoma, lung cancer mimic, melanoma of unknown primary, primary pulmonary melanoma

## Abstract

Melanoma of unknown primary (MUP) with endobronchial involvement is a rare diagnostic presentation that may clinically and radiologically mimic primary lung cancer. We report the case of a 46-year-old man, a chronic smoker with a 20-pack-year history and a previous excisional biopsy of an atypical scalp nevus, who presented with low-volume hemoptysis and constitutional decline evolving over three months. Chest imaging revealed a large necrotic mass involving the right middle and lower lobes, associated with bilateral pulmonary nodules in a cannonball pattern and mediastinal lymphadenopathy, initially suggesting an advanced primary lung malignancy. Bronchoscopy demonstrated an obstructing endobronchial lesion at the level of the bronchus intermedius. Histopathological examination revealed a malignant melanocytic proliferation, with immunohistochemistry showing positivity for melanoma antigen recognized by T cells 1 (Melan-A) and human melanoma black 45 (HMB-45) and negativity for thyroid transcription factor-1 (TTF-1), cytokeratin AE1/AE3 (AE1/AE3), and p40, supporting the diagnosis of malignant melanoma. Extensive evaluation for an extrathoracic primary, including complete dermatological and mucosal examination and fundoscopy, failed to identify a primary lesion. Staging revealed additional brain, peritoneal, and bone involvement at diagnosis. Primary pulmonary melanoma and MUP with endobronchial involvement were considered, with the overall clinical and pathological findings favoring the latter diagnosis. The case was discussed at a multidisciplinary tumor board, and systemic immunotherapy was initiated. This case highlights the diagnostic challenge posed by an endobronchial melanocytic lesion in a patient with a smoking history and emphasizes the importance of a thorough search for an extrathoracic primary before diagnosing primary pulmonary melanoma.

## Introduction

More than 90% of melanomas arise in the skin [1]. A small proportion originate at other sites, including mucosal surfaces, the uvea, and the meninges [2]. The lung, however, is predominantly a site of metastatic involvement, and pulmonary melanoma is secondary in the vast majority of cases [3-6]. Endobronchial involvement is itself an uncommon presentation, most often reported as a metastatic deposit, with primary endobronchial melanomas described only sporadically [7].

Primary pulmonary melanoma is a distinct but exceptionally rare entity, with fewer than 50 cases reported in the English-language literature since its first description in 1916 [3-6]. Its diagnosis relies on strict clinical exclusion criteria, originally attributed to Jensen and Egedorf and detailed in subsequent literature reviews [3,8], together with the histopathological criteria described by Allen et al. [3,9]. These criteria emphasize the exclusion of an extrapulmonary primary and, histologically, the presence of junctional/intraepithelial melanocytic changes adjacent to an invasive melanoma. A comprehensive clinical, dermatological, and ophthalmological evaluation is therefore required to exclude an extrapulmonary primary. When no primary site can be identified, particularly in the setting of disseminated disease at presentation, melanoma of unknown primary (MUP) with pulmonary or endobronchial involvement should be considered [10,11]. This distinction is clinically relevant, although differentiating between the two entities can be challenging. Endobronchial presentation of MUP remains exceptionally rare, with only a limited number of cases reported to date [7].

Radiologically, these entities may closely mimic primary lung cancer, presenting as a pulmonary mass with or without multiple bilateral nodules in a "cannonball" pattern [3,5]. In a chronic smoker, such findings may strongly suggest bronchogenic carcinoma-and this was, indeed, the leading hypothesis in our own patient before pathology proved otherwise. We report this case to highlight the diagnostic challenge of an endobronchial melanocytic lesion, detail the reasoning that favored MUP over primary pulmonary melanoma, and discuss it in the context of the available literature.

## Case presentation

A 46-year-old man, a chronic smoker with a 20-pack-year history and ongoing tobacco use at presentation, was referred to our department for low-volume hemoptysis. He had no relevant family history of melanoma or skin cancer. His only notable medical history was an excisional biopsy of an atypical scalp nevus approximately 17 years before presentation.

The patient's symptoms had begun approximately three months before admission with a productive cough that progressively became blood-streaked. The course was afebrile but associated with constitutional symptoms, including asthenia, anorexia, and a 5-kg weight loss over three months. He also reported right-sided lower back pain of inflammatory type, predominantly nocturnal, without any history of trauma.

On admission, the patient was pale but alert and breathing comfortably on room air, with a preserved performance status (Eastern Cooperative Oncology Group performance status 1). Physical examination revealed clinical signs of right basal pulmonary consolidation, without evidence of pleural effusion. The remainder of the examination, including inspection of the skin, was unremarkable.

Laboratory investigations performed on admission revealed an inflammatory syndrome, with normocytic anemia (hemoglobin 12 g/dL), neutrophil-predominant leukocytosis (13,000 cells/mm³), and an elevated C-reactive protein level (50 mg/L). Renal and liver function tests were within normal limits.

Chest radiography performed during the initial evaluation showed a right paracardiac, polylobulated opacity associated with multiple nodular opacities scattered throughout both lung fields. Chest computed tomography (CT) subsequently revealed a relatively well-defined mass involving the right middle and lower lobes, measuring 75 × 82 × 62 mm, with heterogeneous attenuation due to central necrotic areas. Multiple bilateral pulmonary nodules of variable size produced a characteristic "cannonball" pattern. Mediastinal lymphadenopathy was limited to a 4R lymph node measuring 11 mm in short axis (Figure 1).

**Figure 1:**
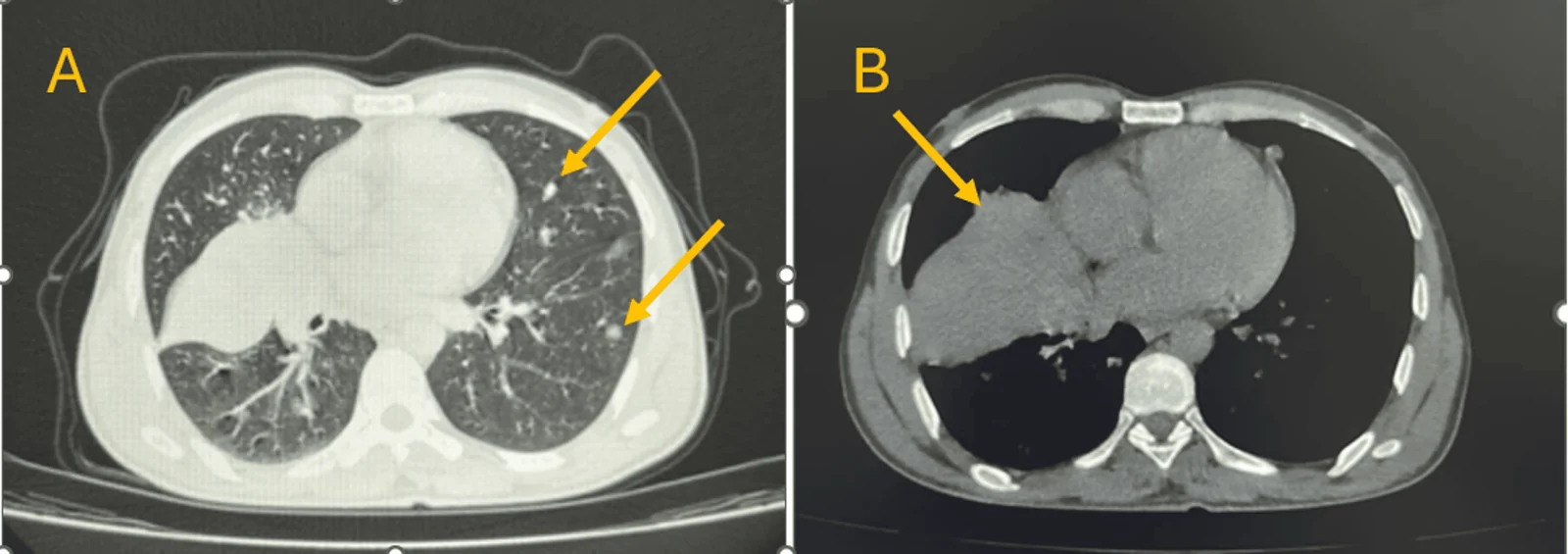
Chest CT showing the pulmonary mass and multiple pulmonary nodules (A) Axial CT image in the lung window showing a well-defined pulmonary mass in the right lower lobe, associated with multiple bilateral pulmonary nodules (yellow arrows). (B) Axial CT image in the mediastinal window showing the same lesion as a right hilar mass (yellow arrow).

Bronchoscopy performed during the initial diagnostic workup revealed a grayish, friable endobronchial mass partially obstructing the bronchus intermedius (Figure 2). Multiple biopsies were obtained. Histopathological examination showed a diffuse malignant tumor proliferation composed of large atypical cells with clear cytoplasm and eccentric nuclei, associated with intracytoplasmic melanin pigment. Immunohistochemical staining demonstrated strong and diffuse positivity for melanoma antigen recognized by T cells 1 (Melan-A) and human melanoma black 45 (HMB-45), whereas thyroid transcription factor-1 (TTF-1), cytokeratin AE1/AE3 (AE1/AE3), and p40 were negative. This immunophenotypic profile supported the diagnosis of malignant melanoma and argued against an epithelial bronchial malignancy, including adenocarcinoma and squamous cell carcinoma.

**Figure 2:**
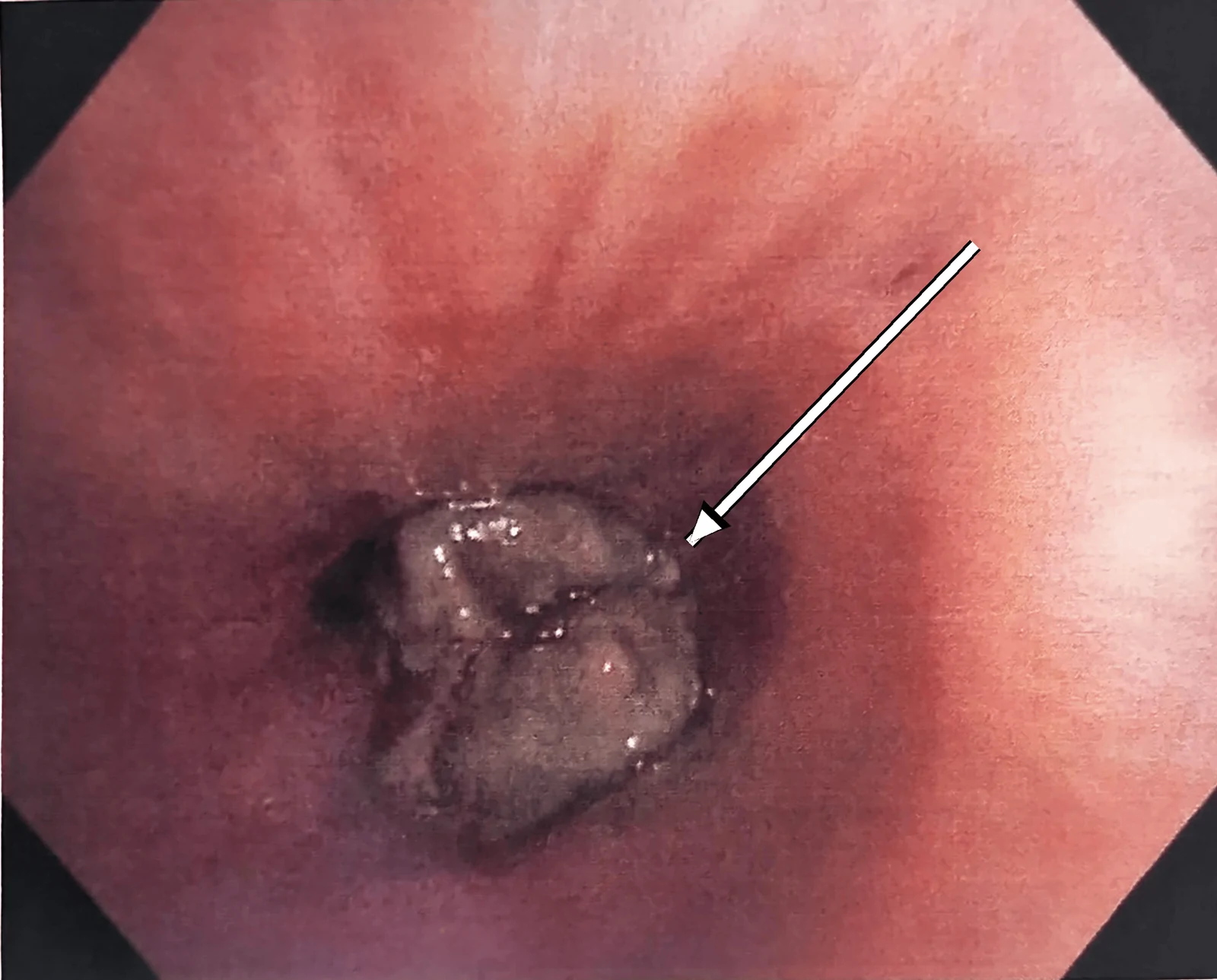
Bronchoscopic appearance of a grayish endobronchial mass obstructing the bronchus intermedius

An extensive search for a primary melanoma was subsequently performed. Complete examination of the skin and mucosal surfaces, including the scalp, oral and genital mucosa, and anorectal region, revealed no suspicious primary lesion. Fundoscopic examination was normal, providing no evidence of an ocular primary (Figure 3).

**Figure 3:**
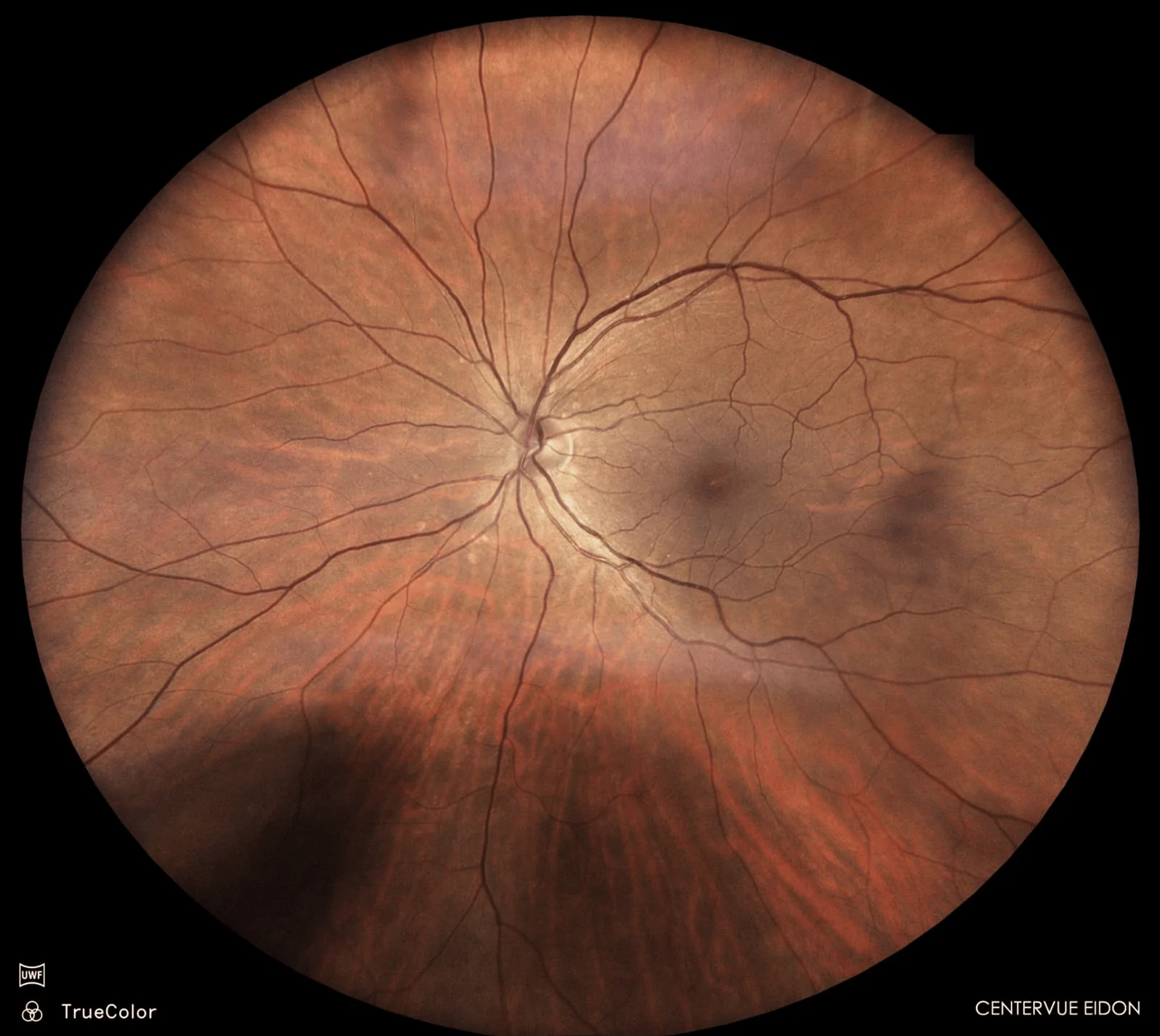
Color fundus photograph of the right eye (OD), showing no evidence of uveal melanoma

Staging investigations subsequently demonstrated disseminated disease, including a 12-mm left temporal brain lesion consistent with metastasis (Figure 4), multiple peritoneal implants, and two suspicious bone lesions involving the L1 vertebral body and right femoral head, which showed increased radiotracer uptake on bone scintigraphy. These findings provided a plausible explanation for the patient's lower back pain at presentation. No FDG PET/CT was performed.

**Figure 4:**
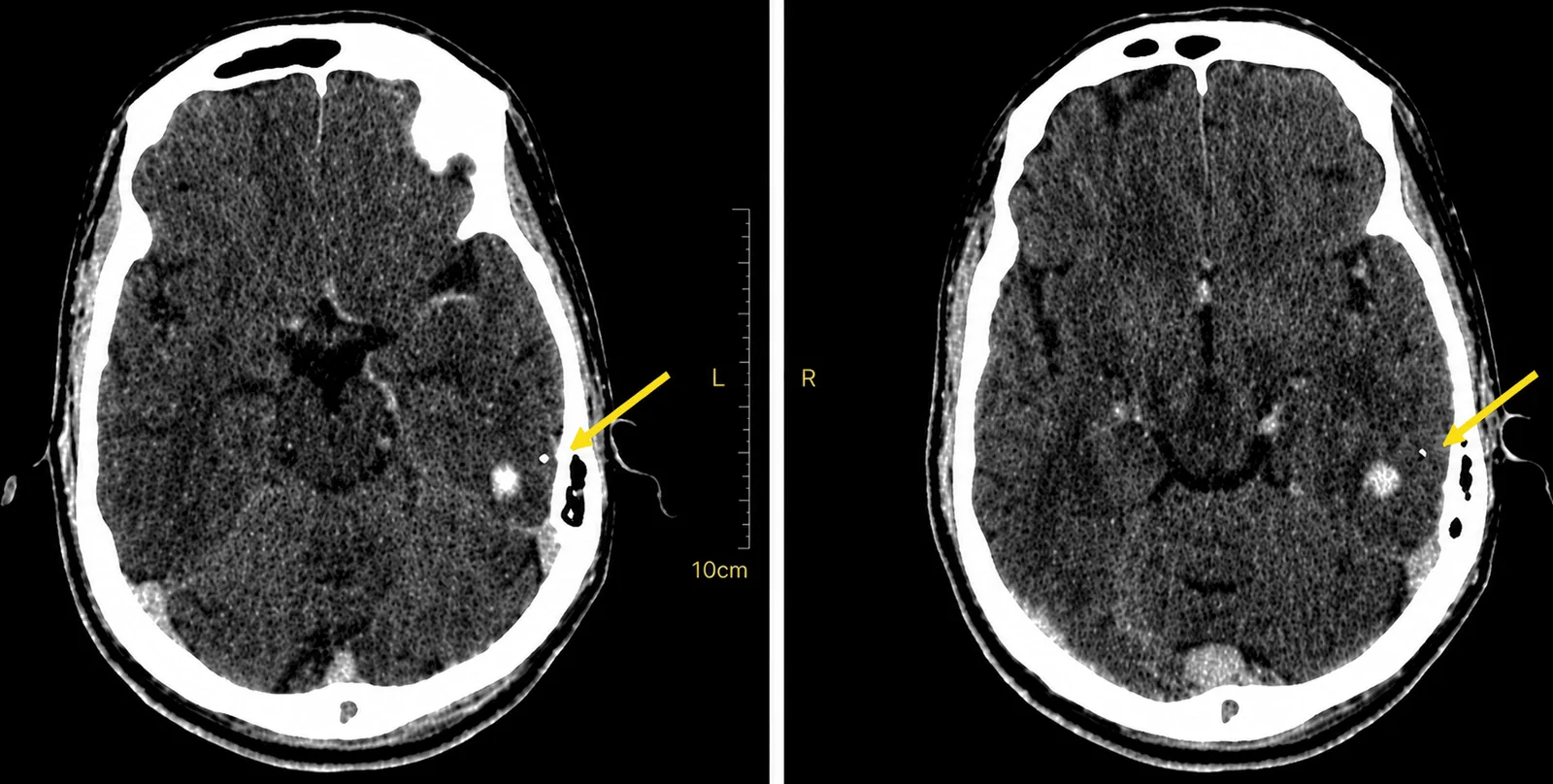
Contrast-enhanced axial brain computed tomography showing a hyperdense focal lesion in the left temporal region (yellow arrows)

The case was discussed at a multidisciplinary thoracic oncology tumor board. Given the absence of a cutaneous, mucosal, or ocular primary, the findings favored endobronchial MUP with metastatic disease (stage IV). The patient was considered a candidate for anti-PD-1 immunotherapy, and a second percutaneous biopsy was performed to obtain additional tissue for molecular characterization and therapeutic planning. The procedure was complicated by pyopneumothorax and subsequent septic shock. Despite appropriate management, the patient died during the same hospitalization before immunotherapy could be initiated.

## Discussion

MUP with endobronchial presentation lies at the intersection of two distinct pathological processes - primary pulmonary melanoma and metastatic MUP - for which the available literature remains limited to a small number of published cases [3-7]. Our observation is discussed below with regard to patient background, radio-clinical and endoscopic characteristics, diagnostic approach, treatment, and prognosis reported in the literature.

The incidence of MUP is estimated at approximately 3% of all melanomas, across all stages [10,11]; an endobronchial presentation of MUP remains exceptional, with the available literature limited to isolated cases [7]. In terms of patient background, our patient stands out for his age: 46 years, compared with a mean age of 57-59.1 years in the largest published series of primary pulmonary melanoma [3,6], with ranges of 29-90 years [3] and 41-82 years [6]. A recently published case, however, reported an index case of the same age (46 years) [5], suggesting that this age group may be underrepresented in historical series rather than genuinely spared by the disease. Chronic smoking (20 pack-years) is not a recognized risk factor for melanoma, but it points, in the first instance, toward bronchogenic carcinoma: it was precisely this background bias that initially sustained the hypothesis of a primary bronchopulmonary cancer in our patient, before histopathology refuted it.

Radioclinically, the picture observed in our patient - a large, necrotic pulmonary mass, bilateral nodules producing a "cannonball" appearance, mediastinal lymphadenopathy - is superimposable on that reported in series of primary pulmonary melanoma and in published cases of MUP with pulmonary presentation [3-7]. Endoscopically, the appearance of a grayish tumor mass obstructing a proximal bronchus, found here in the bronchus intermedius, is also described in the case of Kim et al., where the mass obstructed the lingular bronchus [7]. This radio-endoscopic similarity accounts for the diagnostic difficulty specific to this presentation: neither imaging nor bronchoscopic appearance alone can distinguish a primary pulmonary melanoma from an endobronchial localization of MUP. Only histological analysis, complemented by immunohistochemistry, can establish the melanocytic nature of the lesion; in our patient, positivity for Melan-A and HMB-45 combined with negativity for TTF-1, AE1/AE3, and p40 formally excluded an epithelial bronchial origin.

It is precisely at this stage that the diagnostic difficulty persists, and where our observation offers some insight: even after histological confirmation of the melanocytic nature of the tumor, the distinction between primary pulmonary melanoma and endobronchial MUP rests on indirect criteria rather than on a specific histological marker. Two hypotheses were weighed:

Hypothesis one: Primary pulmonary melanoma: arguments in favor - a dominant, bulky pulmonary mass, a substantial endobronchial component, absence of a cutaneous, mucosal, or ocular primary despite thorough search, an immunohistochemical profile consistent with melanocytic differentiation; arguments against - diffuse metastatic disease at diagnosis (brain, peritoneum, bone), the absence, in the histopathological description, of a junctional or in situ component supporting a primary bronchial origin, an element required by the Allen-Drash histopathological criteria [3,9], and the presence of multiple extrapulmonary sites.

Hypothesis two: MUP with endobronchial localization: arguments in favor - histologically confirmed melanoma, negative cutaneous, mucosal, and ophthalmological work-up, multisite metastatic dissemination, possible spontaneous regression of a cutaneous primary, the lung being a documented site of melanoma metastasis; limitation - the bulky pulmonary mass with a dominant endobronchial component can still raise the possibility of a primary pulmonary origin. The absence of a junctional component on histopathology and the extent of extrapulmonary disease tip the balance toward the second hypothesis, without entirely excluding the first.

A third hypothesis, specific to our observation, must be incorporated into this reasoning: the history of an atypical nevus of the scalp, excised in 2009 (17 years before the current presentation) and read as benign at the time, whose original slides and report could not be retrieved for review. This lesion could itself have corresponded to an unrecognized primary melanoma at the time, in which case the current presentation would represent an ultra-late metastatic recurrence of a known melanoma rather than a MUP. This hypothesis is consistent with the literature: an estimated minimum of 2% of melanoma patients experience their first recurrence at 15 years or beyond, with an average disease-free interval of 17.3 years in women and 20.0 years in men in a landmark single-center series of ultra-late recurrences [12]. A separate single-center series of patients with melanoma recurrence 10 years or more after initial diagnosis similarly documents this phenomenon of prolonged tumor dormancy [13]. The 17-year interval observed in our patient falls within the range reported for ultra-late recurrence. In the absence of the original pathology material, this hypothesis cannot be confirmed; it constitutes a limitation of this work and underscores that a distant dermatological history cannot be dismissed without histological review.

Therapeutically, the strategy adopted depends directly on the extent of disease at diagnosis, independent of the nosological question. In the localized endobronchial MUP case described by Kim et al., curative surgical resection was performed, the disease being confined to the bronchus at presentation, although recurrence occurred at four months [7]. In our patient, multi-organ involvement at presentation (brain, peritoneum, bone) precluded any curative surgical option and justified systemic immunotherapy. This difference in management illustrates the importance of complete staging - rather than local biopsy alone - in guiding treatment.

Prognostically, the available data for metastatic MUP have been profoundly changed by the advent of immunotherapy. Before the era of immune checkpoint inhibitors, five-year overall survival for metastatic MUP was estimated at between 8% and 18% [14]. A retrospective American cohort study of patients with advanced melanoma treated between 2014 and 2020 (n = 79) reported, among patients with unknown primary, a three-year overall survival of 92% versus 62% for patients with known primary, and at five years, 83% versus 44% [15,16] - figures markedly higher than the pre-immunotherapy range cited above, though drawn from a small single-center cohort. A Dutch population-based study of 2,706 patients [17] reports a significant improvement in overall survival among MUP patients in the era of targeted therapy and immunotherapy, with a slight advantage over patients with known primary after adjustment, despite a more unfavorable presentation at baseline (more than half of patients at stage four at diagnosis) [17]. This prognostic improvement may be related, in a subset of MUP patients, to spontaneous regression of the primary cutaneous tumor mediated by a T-cell and natural killer cell immune response [18], reflecting a more reactive host immune background potentially more responsive to a therapeutic strategy based on restoring anti-tumor immunity [18]. These data directly informed the therapeutic choice made by our team.

Our observation contributes three elements to the existing literature, beyond the case itself. First, it documents an additional case of MUP with endobronchial presentation, an entity for which the literature to date includes only rare published cases [7]. Second, it concretely illustrates the diagnostic difficulty posed by an endobronchial melanocytic mass in a smoker, where the smoking background, radiological appearance, and endoscopic appearance all converge on a false lead of bronchogenic carcinoma, delaying consideration of melanocytic hypotheses until immunohistochemistry is obtained. Third, through the unreviewed atypical nevus history, it highlights the importance of preserving and making accessible the pathology material of excised skin lesions, the only means of distinguishing between MUP and ultra-late recurrence of an initially mislabeled melanoma.

## Conclusions

This case illustrates a genuine diagnostic dilemma: an endobronchial melanocytic mass in a smoker, initially suggestive of a primary pulmonary malignancy, ultimately favored MUP after comprehensive staging revealed multi-organ dissemination. The diagnostic uncertainty was further compounded by a scalp nevus excised 17 years earlier, whose original pathology could not be re-examined and which may have represented a previous primary melanoma. Given the rarity of endobronchial MUP, careful application of established diagnostic criteria is essential when considering a primary pulmonary origin. Although modern immunotherapy has improved outcomes in metastatic melanoma, our patient died from septic shock following an invasive diagnostic procedure before immunotherapy could be initiated, underscoring both the diagnostic complexity and the potential risks of invasive procedures in advanced disease.
